# Teaching Self-Regulated Learning Through Reflective Writing: Experiences of First-Year Medical Students With the Master Adaptive Learner Model

**DOI:** 10.7759/cureus.72664

**Published:** 2024-10-29

**Authors:** Shawn Koh, Jonathan Townsend

**Affiliations:** 1 Department of Medical Education, California University of Science and Medicine, Colton, USA

**Keywords:** innovation in medical education, master adaptive learner, medical education, medical education curriculum, medical education research, reflection essay, self-regulated learning, undergraduate medical student

## Abstract

Background: In recent years, the Master Adaptive Learner (MAL) model has received widespread attention as a self-regulated learning (SRL) model in medical education. Yet, little research has been done on exploring the benefits of explicitly teaching this model to undergraduate medical students.

Methods: We taught this model in a required social science course to first-year medical students at the California University of Science and Medicine. Education was delivered via three two-hour sessions, each involving one hour of didactic presentation followed by one hour of small group discussion. Students then applied the model through a series of six structured reflection essays. After this intervention, we deployed a survey to understand student responses to using the MAL model.

Results: The majority of respondents indicated that the MAL model increased their self-awareness and intentionality in learning. Students also attested that it increased the depth and breadth of learning about self-identified learning gaps.

Conclusion: This study suggests that the MAL is an effective SRL model to promote metacognition and depth of learning in undergraduate medical students.

## Introduction

Self-regulated learning (SRL)

Physicians are problem-solvers. They identify a problem, gather necessary data, and use clinical reasoning to determine a diagnosis and therapy. Much of what successful clinicians do requires that they be exceptional learners. The study of how people learn has deep historical roots and can be traced back to the philosophical inquiries of Plato and Aristotle. However, the emergence of cognitive psychology in the 1950s marked a significant shift, positioning cognitive and metacognitive processes as central to understanding how learning works [1]. Researchers have amassed a corpus of work describing the processes and strategies that effective learners use, and many studies have found that effective learners engage in self-regulatory strategies that support and enhance learning [2-5]. SRL strategies are a vital component of successful learning, and perhaps most importantly, SRL strategies can be taught and learned [3,5]. It is valuable to situate SRL instruction within discipline-specific contexts; teaching SRL strategies and tools in context allows learners to immediately apply them. [2,3,5]. 

SRL and the Master Adaptive Learner (MAL) model

SRL was defined by Zimmerman and Schunk as “...the processes whereby learners personally activate and sustain cognitions, affects, and behaviors that are systematically oriented toward the attainment of personal goals” [6]. Foundational research from scholars in educational psychology like Zimmerman, Pintrich, and Pajares described the construct of SRL as having domains and phases [2,7,8]. According to Zimmerman and Schunk, there are four domains of SRL: the cognitive and metacognitive domain, the developmental domain, the motivational domain, and the social and environmental domain [6]. The SRL process can occur in any of these four domains. 

Zimmerman described the SRL process as having three phases: the forethought phase, the performance phase, and the self-reflection phase. The forethought phase includes elements of task analysis and self-motivation beliefs. The performance phase includes the elements of self-control and self-observation. The self-reflection phase includes elements of self-judgment and self-reaction [9]. In contrast, Winne and Hadwin described SRL as having four phases, namely 1) gathering information and personalizing their perception of the information, 2) goal-setting and planning, 3) enacting their plan, 4) adaptation, including modification of the approach for the application of a new cycle of learning [4]. While the study of SRL was initially centered in the field of educational psychology, its use as an academic tool expanded to other applied educational fields such as medical education. 

Several studies have explored the role of SRL in effective medical education. In one example, students who utilized SRL tools like concept-mapping and goal-setting demonstrated more confidence, control and metacognition in problem-based learning [10]. Conversely, another study revealed that students who excelled in medical knowledge reported using a variety of SRL strategies [11]. This same trend was seen in graduate medical education (GME), where residents identified by program leadership as exceptional learners used various elements of planning, an element of SRL, in their learning [12]. 

SRL strategies are utilized in various phases and contexts in medical education. In medical schools with integrated, student-centered curricula, first-year medical students utilized a wide variety of SRL strategies [13]. Similarly, clerkship students use a wide variety of SRL strategies, with evidence showing that the tools utilized varied with the learning context [14]. It still remains unclear what proportion of medical students consistently use SRL strategies in learning, and indeed, some data suggest that their use of these skills is limited in consistency and breadth when the approaches are not explicitly taught [15]. Reassuringly, when explicitly taught as early as the first year of medical school, students show an increased motivation in using SRL skills for future learning [16]. 

In recent years, the American Medical Association has disseminated the MAL model, an adaptive learning framework that incorporates metacognition and SRL strategies into four phases. Learners begin with the planning phase, which involves recognizing a learning gap, prioritizing the scope of learning, and identifying appropriate resources. During the learning phase, they explore and internalize source material using evidence-based learning strategies and critically appraise the strength of evidence behind each new idea. They then assess their learning by trying out the concepts learned and reflecting on how well the learning gap was closed. A key to this phase is the integration of self-assessment with external feedback. Finally, the adjusting phase involves the implementation of the new learning into their daily routine, which may include individual and/or systemic levels of application [17].

The MAL model specifically articulates its purpose as developing clinicians to be effective and self-regulated master learners [17]. This model is highly congruent with both Zimmerman’s (2008) and Winne and Hadwin’s (2008) models of SRL, although the MAL model includes less granularity. The MAL model’s phases of planning, learning, assessing, and adjusting include fundamental components of both Zimmerman’s [6] and Winne and Hadwin’s [4] models, but the latter models are more comprehensive than the former and provide detailed descriptions for several factors in each of the SRL phases. Despite these differences, the MAL model contains core SRL concepts such as forethought and planning (the planning phase) and self-reflection (assessing phase). The MAL model, by its design, is not as comprehensive as other SRL models. It is a tool for a specific context but, nonetheless, its core components are rooted deeply in SRL. 

To our knowledge, there has been little published data on the methods to explicitly teach MAL or measure its effectiveness in learning. In this study, we taught the MAL model to first-year medical students through three class sessions and a series of six reflection essays structured around the model's four SRL phases. Our research objectives were to examine the impact of the MAL model on student learning processes and to examine their perceptions on the effectiveness of this approach in facilitating the exploration of their learning gap. 

This study was previously delivered as an oral poster presentation at the 2024 Association of American Medical Colleges Western Group on Educational Affairs Spring Conference on May 6, 2024.

## Materials and methods

The MAL model was taught to first-year medical students through three sessions in a year-long medical humanities and social science course. Each session involved one hour of didactic education followed by one hour of small group discussion. In the first session, students were introduced to the physician experience of clinical surprises, when a routine approach to patient care does not achieve the desired outcome. This highlighted the necessity of intellectual humility and curiosity in medical practice. The session then presented the MAL model (including its planning, learning, assessing, and adjusting phases) as a process for addressing learning gaps. After this initial session, students were introduced to their year-long assignment. They were asked to choose an individual social science learning gap and document their exploration through a series of six structured reflection essays modeled after the MAL process. The social science learning topic constraint was included because it aligned with the course and we thought it would encourage the selection of a gap that required an adaptive learning approach.

The first two papers, the first formative and the second summative, centered around the planning phase. Students were asked to identify a gap, justify their motivation, and describe their plan for exploration. After formative feedback on the first paper, students had the opportunity to refine their planning phase reflection in a second paper that was scored. The third paper, centered on the learning phase, asked students to summarize their learning from at least three sources and discuss the validity and reliability of each source. Then students engaged in the second session on the MAL model, which taught the assessing phase and the value of high-quality feedback. They subsequently wrote the fourth paper assessing their effectiveness in closing their learning gap. This paper included the integration of self-reflection and feedback obtained from three people, including at least one subject matter expert. The final instructional session on the MAL model, based on the adjusting phase, taught students to consider both individual and systemic applications of their learning. Students then wrote the fifth paper identifying ways to apply what they learned to individual and systemic settings. The sixth and final paper was a summary of the students' experience with their learning gaps, a critical reflection on the strengths and limitations of using the MAL model, and additional learning-gap-associated questions for future exploration.

At the end of the academic year, we initially designed a qualitative methodology using focus groups to study the student experience with the MAL model. Unfortunately, since only two students enrolled to participate in focus groups, we did not proceed with this method and instead adjusted our methodology from the use of focus groups to a survey instrument. While grading the reflection papers, the course director (and first author of this article) noticed some themes in student reflections on the effectiveness of the MAL model in guiding their learning. These themes were used to design a brief survey to quantify student perceptions related to these five themes. The authors of this study received approval from the IRB of California University of Science and Medicine to conduct this quantitative study. The survey consisted of five Likert-style questions and two short-response questions (Table 1). 

**Table 1:** Survey questions used to assess student perceptions of the Master Adaptive Learner (MAL) model

Survey Questions
The MAL model reduced the emotional load of learning about my gap
The MAL model helped me to be more self-aware and intentional about my learning process
The structure of the MAL model facilitated more comprehensive or deeper learning on my gap than I would have achieved without the model
The MAL model increased the value I place on external feedback in driving my learning
I plan to use the MAL model for future learning
Strengths of the MAL model
Limitations or weakness of the MAL model

## Results

Quantitative results

**Figure 1:**
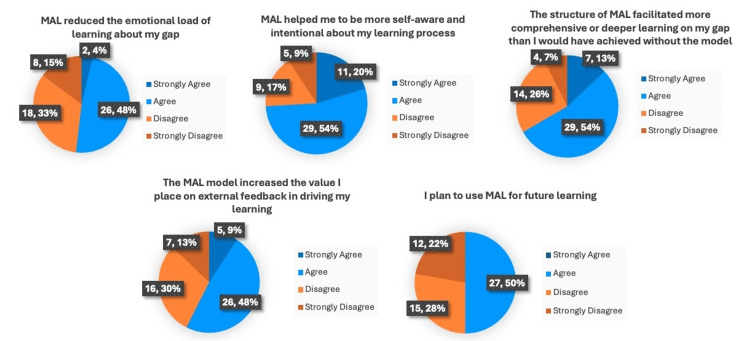
Students' (n=54) perceptions of the impact of the Master Adaptive Learner (MAL) model on learning (frequency, percent)

Out of the 129 students in the course, 54 (42%) consented to participate in and complete the survey. A majority of respondents reported that using the MAL model helped increase their self-awareness and intentionality about their learning process (n=40, 74%) and noted that it facilitated more comprehensive or deeper learning about their gap than they would have achieved without it (n=36, 67%). Though several students indicated that the model reduced the emotional load of learning about their gap in their final reflection paper, only about half (n=28, 52%) of the survey respondents reported this. A slightly greater proportion (n=31, 57%) felt that the MAL model increased the value they placed on external feedback in their learning. Only half of them (n=27, 50%) planned to use it for future learning (Figure 1).

Qualitative results

While this study design emphasized quantitative data, the students’ short answer responses regarding the strengths and limitations of the MAL model provided some additional qualitative insight for future exploration. Some of the salient responses are included below.

Strengths of the MAL Model

“It provides focused steps to make learning easier.”

“It is a step-wise, logical, algorithmic approach.”

“Took a daunting task and broke it down into more manageable chunks.” 

Limitations of the MAL Model

“It is common sense… MAL did not teach me anything I didn’t already know.” 

“The amount of steps done seemed too many for the subject matter I chose.”

“Requires reflection, self-awareness, and most importantly a desire to actually know, care, and implement change little by little.”

## Discussion

This study contributes to the limited data on the impact of explicitly teaching the MAL model to undergraduate medical students. Our study led us to three conclusions. Firstly, it revealed that it is feasible to teach the MAL model to medical students in a way most understood and found helpful. Specifically, students can be taught to engage in SRL by exploring social science topics through a series of structured reflection essays. Many US medical schools include a social science course in their curriculum, and this model of structured reflection essays could be feasibly integrated into these courses. Secondly, the structured reflection essays provided a scaffolding for SRL, facilitating increased metacognition and ultimately deeper learning about a learning gap. Third, some students concluded that the MAL model, at least when applied through a series of reflection essays, was of limited value. Only half of the respondents expressed that they would be likely to use this model for future learning. This invites exploration of the limitations the students associated with the model. 

The short answer comments provided us with some depth regarding student experiences. Several students remarked that the model was common sense, or intuitive, and added little to what they already knew. This may indicate that some students have already learned and integrated self-regulatory processes in their learning routines. It is worth noting that a study of first-year Harvard medical students showed that less than half the class routinely used SRL tools like goal-setting and reflection at the start of medical school [16]. It is likely, therefore, that the majority of medical students could benefit from explicit SRL instruction. The participants’ lack of enthusiasm for using MAL for future learning could also reveal that students did not recognize the key benefits of SRL or were unclear about how SRL could be applied to other types of learning, like basic and clinical science topics. 

Another possible implication of the data is that the MAL process was unnecessarily complex for some student learning gaps. It is notable that the initial authors of the MAL model described it as being particularly useful for gaps that require innovative problem-solving associated with adaptive expertise [17]. Perhaps some of the learning gaps students chose were adequately closed with a simple routine learning process, making this model unnecessarily cumbersome. Some students may also have selected topics that they lacked adequate motivation to pursue as comprehensively as the reflection essay series required. These students may have lacked the passion required to pursue their learning topics with grit [18]. These questions require further study with an exploratory, grounded theory approach. 

Our study had several limitations. We had a relatively low response rate, which introduces the potential for selection bias. Nonetheless, it is still helpful to highlight student responses to a model with little empirical evidence. A second limitation was the use of a quantitative method, which lacked the depth of exploration on student learning processes, the relative strengths and limitations of the MAL model, and their reflections on our method of teaching the model. A future qualitative study could better capture these data. Finally, our survey focused on the students' perception of learning rather than measuring the learning that occurred. Since effective learning is often effortful and uncomfortable, future research on this topic could benefit from the inclusion of a direct measurement of learning outcomes. Further exploration of the MAL model could also occur with different levels of learners, including clerkship students and residents. It would also be helpful to follow-up with our cohort of students and encourage the use of this model in a more rapid-cycle learning, rather than the year-long design utilized in this study. 

## Conclusions

The MAL model is a problem-solving approach rooted in self-regulated learning principles and intended to support a widespread audience of medical students, clinicians, and educators. This study suggests a feasible method of teaching the MAL model to first-year medical students. While most students acknowledged its effectiveness in increasing their metacognition and depth of learning, only half of them saw it as a valuable tool for future learning. This complexity invites future exploration, including a qualitative study of student experiences with the model. Further research is needed to explore the effectiveness of teaching the MAL model in undergraduate and graduate medical education settings, and further research is also needed to understand how medical students and physicians regulate their own learning. 
